# Association of Estimated Glucose Disposal Rate With Risk of Abdominal Aortic Aneurysm: Evidence From a Large-Scale Prospective Cohort Study of the UK Biobank

**DOI:** 10.31083/RCM36776

**Published:** 2025-07-21

**Authors:** Yuanwei Chen, Ting Zhou, Songyuan Luo, Jizhong Wang, Fan Yang, Yingqing Feng, Lixin Fang, Jianfang Luo

**Affiliations:** ^1^School of Medicine, South China University of Technology, 510006 Guangzhou, Guangdong, China; ^2^Department of Cardiology, Guangdong Provincial People's Hospital (Guangdong Academy of Medical Sciences), Southern Medical University, 510080 Guangzhou, Guangdong, China; ^3^Department of Emergency and Critical Care Medicine, Guangdong Provincial People's Hospital (Guangdong Academy of Medical Sciences), Southern Medical University, 510080 Guangzhou, Guangdong, China; ^4^Department of Cardiology, Hypertension Research Laboratory, Guangdong Cardiovascular Institute, Guangdong Provincial People's Hospital, Guangdong Academy of Medical Sciences, Southern Medical University, 510080 Guangzhou, Guangdong, China; ^5^Department of Cardiovascular Medicine, Guangdong Provincial Key Laboratory of Coronary Heart Disease Prevention, Guangdong Cardiovascular Institute, Guangdong Provincial People's Hospital (Guangdong Academy of Medical Sciences), Southern Medical University, 510080 Guangzhou, Guangdong, China

**Keywords:** abdominal aortic aneurysm, estimated glucose disposal rate, insulin resistance, predictive performance

## Abstract

**Background:**

Insulin resistance has been recognized as a risk factor in the pathogenesis of various diseases. The estimated glucose disposal rate (eGDR) has been widely validated as a reliable, noninvasive, and cost-effective surrogate measure of insulin resistance. However, the relationship between eGDR and abdominal aortic aneurysm (AAA) has not yet been fully elucidated. This study sought to investigate the association between the eGDR levels and the risk of AAA development.

**Methods:**

This prospective cohort study enrolled participants from the UK Biobank who had complete eGDR measurements and no pre-existing AAA at baseline (2006–2010). Participants were stratified into quartiles according to their eGDR values. The association between eGDR and AAA was assessed using Cox proportional hazards models with results expressed as the hazard ratio (HR) and 95% confidence interval (CI). Kaplan–Meier curves were generated to visualize cumulative AAA incidence across eGDR quartiles, whereas restricted cubic splines (RCSs) were applied to characterize the exposure–response relationship. Sensitivity and subgroup analyses were conducted to assess the robustness of the findings.

**Results:**

The final analytical cohort comprised 416,800 participants (median age: 58.0 years (IQR: 50.0–63.0), 45.83% male). During the median follow-up of 13.6 years, 1881 incident AAA cases were recorded. The Kaplan–Meier curve analysis demonstrated a higher cumulative AAA risk with decreasing eGDR quartiles (log-rank *p* < 0.05). The Multivariable Cox model confirmed that lower eGDR levels were significantly associated with increased AAA risk. When eGDR was assessed as categorical variable, compared with the participants in Quartile 1 group (reference group), the adjusted HR (95% CI) for those in the Quartile 2–Quartile 4 groups were 0.76 (0.66–0.87), 0.69 (0.59–0.80), and 0.46 (0.35–0.62), respectively. When eGDR was evaluated as a continuous variable, a 1-unit increment in eGDR corresponded to a 12% reduction in AAA risk (HR: 0.88, 95% CI: 0.85–0.90). After excluding patients with pre-existing diabetes or short-term follow-up, the sensitivity analysis produced similar results. A subgroup analysis further maintained the association between eGDR and AAA. Furthermore, the RCS curve revealed a nonlinear association between eGDR and AAA incidence risk (*p* for nonlinearity ≤ 0.05), identifying a threshold value of 7.78.

**Conclusions:**

Our study demonstrates that reduced eGDR levels are independently associated with elevated AAA risk, exhibiting a nonlinear dose–response relationship characterized by a threshold effect at 7.78. These findings position eGDR as a potentially valuable biomarker for AAA risk stratification and interventional strategies.

## 1. Background

Abdominal aortic aneurysm (AAA) is defined as the irreversible and perpetual dilatation of the infrarenal aorta [1]. Globally, AAA accounts for 167,200 annual deaths and results in 3 million disability-adjusted life years lost [2]. The insidious nature of AAA progression, typically remaining asymptomatic until rupture occurs, poses significant clinical challenges for timely detection. Notably, emerging pathophysiological evidence implicates systemic metabolic dysregulation in AAA pathogenesis, particularly through perturbations in glucose metabolism [3], lipid metabolism [4], amino acid metabolism [5], and more. This evolving understanding underscores the urgency to identify novel metabolic biomarkers linked to AAA progression, which may enable personalized risk stratification and inform targeted preventive interventions.

Estimated glucose disposal rate (eGDR), a validated marker of glucose metabolism, serves as a practical alternative to the euglycemic-hyperinsulinemic clamp gold standard for assessing insulin sensitivity [6, 7]. Over the past two decades, eGDR has demonstrated prognostic value in predicting complications in patients with type 1 diabetes [8, 9, 10, 11, 12]. More recently, large-scale epidemiological studies have established that eGDR is independently associated with cardiovascular events [13, 14, 15]. Despite these advances, a critical gap remains regarding eGDR’s potential relationship with AAA development. To address this knowledge gap, we conducted a population-based prospective cohort study utilizing data from the UK Biobank to investigate the association between eGDR levels and incident AAA risk.

## 2. Methods

### 2.1 Database and Participants

**Fig. 1.**
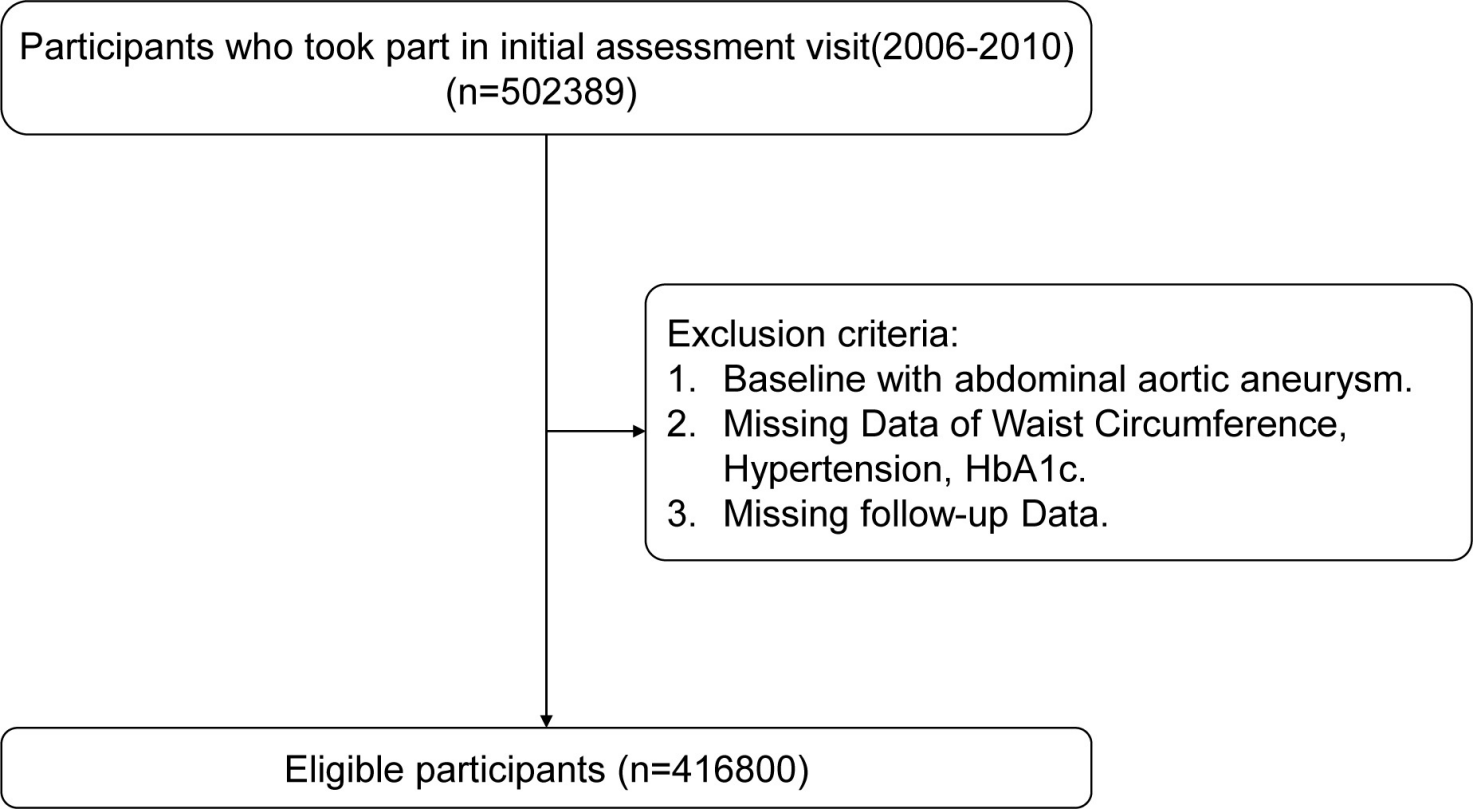
**Flow diagram of participant selection**. HbA1c, glycated hemoglobin.

This study leveraged data from the UK Biobank (2006–2010), a large prospective cohort encompassing approximately 500,000 individuals aged 40–69 years across the United Kingdom. Fig. 1 illustrates a detailed flowchart of the study participants’ selection. From the initial 502,389 participants, we excluded those with AAA at baseline, as well as individuals with missing waist circumference (WC), hypertension (HT) condition, glycated hemoglobin, and missing follow-up data. After applying these exclusion criteria, the analytical cohort comprised 416,800 participants.

### 2.2 Measures

eGDR was calculated using the validated formula: eGDR = 21.158 – (0.09 × WC) – (3.407 × HT) – (0.551× HbA1c) [WC = waist circumference (cm), HT = hypertension status (1 = present, 0 = absent), and HbA1c = glycated hemoglobin] [16]. Participant data retrieved from the UK Biobank included sociodemographic factors (age, sex, ethnicity, education, environment), lifestyle behaviours (smoking, alcohol consumption), and clinical conditions (diabetes, hypertension, coronary heart disease). Anthropometric evaluations included vertical stature, weight assessment, and waist perimeter measurements. Laboratory investigations comprised fasting plasma glucose determination, HbA1c quantification, and detailed serum lipid profiling (measuring total cholesterol fractions, high-density lipoproteins, low-density lipoproteins, and triglyceride levels). We calculated the body mass index (BMI) using mass-to-height ratios (kg/m^2^).

### 2.3 Outcomes

The primary endpoint was defined as incident AAA identified through International Classification of Diseases, Tenth Revision (ICD-10) codes I71.3–I71.4. Patient identification involved comprehensive data collection from three sources: death registries, hospital records, and primary care databases. AAA cases were ascertained through linked records from death registries, hospital admissions, and primary care databases. The earliest recorded diagnosis in any of these sources was considered the time of AAA onset.

### 2.4 Statistical Analysis

For covariates with sparse missing data (<10%), continuous variables were completed using arithmetic means, while categorical variables were supplemented with their most frequent values. Participants were stratified into quartiles according to baseline eGDR levels, with cut-off values determined by the 25th, 50th, and 75th percentiles of the population distribution. Analytical comparisons across these quartiles incorporated the Kruskal-Wallis test for non-parametric continuous data, standard ANOVA for normally distributed variables, and chi-square testing for categorical outcomes.

Kaplan-Meier survival curves were generated to assess cumulative AAA risk, with group differences evaluated using log-rank tests. Cox proportional hazard regression models were constructed to quantify the association between eGDR and AAA risk. The analysis followed a stepwise adjustment protocol: Model 1: adjusted for sex and age; Model 2: further adjusted for drinking and smoking status; and Model 3: additionally adjusted for ethnic, education, BMI, diabetes, coronary heart disease (CHD), fasting blood glucose (FBG), total cholesterol (TC), triglycerides (TG), low-density lipoprotein cholesterol (LDL-C), high-density lipoprotein cholesterol (HDL-C). We conducted separate analyses for each subgroup population using Model 3. Restricted cubic splines (RCS) were implemented to characterize potential nonlinear relationships. Time-dependent ROC curves were generated, and area under the curve (AUC) estimates were calculated to assess eGDR’s discriminative capacity.

Three sensitivity analyses were conducted to assess result robustness: (1) exclusion of participants with prevalent diabetes; (2) exclusion of AAA cases within 1-year follow-up; and (3) exclusion of AAA cases within 3-year follow-up. Schoenfeld residuals served to evaluate the proportional hazards assumption. All analyses were performed using R statistical software (version 4.3.0, RStudio, Inc., Boston, Massachusetts, America), implementing a significance threshold of *p* < 0.05 (two-sided).

## 3. Results

### 3.1 Baseline Characteristics

Table 1 presents the baseline characteristics of the 416,800 participants stratified by eGDR quartiles, including demographic parameters, metabolic profiles, and comorbid conditions. The cohort had a median age of 58.0 years. Participants in lower eGDR quartiles (reflecting greater insulin resistance) demonstrated progressively higher median age. Of the 416,800 study participants, 191,022 (45.83%) were male. The proportion of male participants was inversely correlated with eGDR quartiles, with a higher prevalence in the lower quartiles (*p* < 0.001). Participants in the lower eGDR quartiles exhibited higher glycated haemoglobin levels, increased BMI, larger waist circumference, and elevated fasting glucose and triglyceride levels. These participants also showed lower levels of HDL cholesterol. Additionally, this group exhibited a greater frequency of tobacco use and concurrent medical conditions, encompassing diabetes, elevated blood pressure, and cardiac disease. Statistical analysis (*p* < 0.001) confirmed the significance of these differences.

**Table 1.** **Baseline characteristics of participants**.

Characteristics		Total (n = 416,800)	Quartiles of eGDR				
			Quartile 1 (n = 104,285)	Quartile 2 (n = 104,121)	Quartile 3 (n = 104,244)	Quartile 4 (n = 104,150)	*p* value
eGDR		7.78 [6.16, 10.36]	5.34 [4.65, 5.79]	6.86 [6.51, 7.27]	9.29 [8.46, 9.89]	11.24 [10.79, 11.73]	<0.001
Age, years		58.0 [50.0, 63.0]	60.0 [54.0, 65.0]	60.0 [54.0, 65.0]	56.0 [49.0, 62.0]	53.0 [46.0, 60.0]	<0.001
Male, n (%)		191,022 (45.83)	72,667 (69.68)	49,120 (47.18)	48,813 (46.83)	20,422 (19.61)	<0.001
Ethnic, n (%)							<0.001
	White	396,345 (95.09)	98,792 (94.73)	99,432 (95.50)	98,883 (94.86)	99,238 (95.28)	
	Others	20,455 (4.91)	5493 (5.27)	4689 (4.50)	5361 (5.14)	4912 (4.72)	
Education, n (%)							<0.001
	High	135,348 (32.47)	26,333 (25.25)	30,889 (29.67)	35,706 (34.25)	42,420 (40.73)	
	Medium	210,154 (50.42)	52,817 (50.65)	52,389 (50.32)	53,249 (51.08)	51,699 (49.64)	
	Low	71,298 (17.11)	25,135 (24.10)	20,843 (20.02)	15,289 (14.67)	10,031 (9.63)	
Smoking, n (%)							<0.001
	Yes	190,340 (45.67)	56,892 (54.55)	46,444 (44.61)	46,848 (44.94)	40,156 (38.56)	
	No	226,460 (54.33)	47,393 (45.45)	57,677 (55.39)	57,396 (55.06)	63,994 (61.44)	
Drinking, n (%)							<0.001
	Yes	209,357 (50.23)	50,297 (48.23)	54,299 (52.15)	51,149 (49.07)	53,612 (51.48)	
	No	207,443 (49.77)	53,988 (51.77)	49,822 (47.85)	53,095 (50.93)	50,538 (48.52)	
Environment, n (%)							<0.001
	Urban	357,211 (85.70)	90,563 (86.84)	88,521 (85.02)	89,367 (85.73)	88,760 (85.22)	
	Country	59,589 (14.30)	13,722 (13.16)	15,600 (14.98)	14,877 (14.27)	15,390 (14.78)	
BMI, kg/m^2^		26.75 [24.15, 29.90]	31.06 [28.66, 34.30]	26.43 [24.73, 28.44]	26.76 [24.20, 29.48]	23.70 [22.00, 25.52]	<0.001
Waistline, cm		90.0 [81.0, 99.0]	103.0 [99.0, 110.0]	88.0 [84.0, 92.0]	92.0 [78.0, 98.0]	78.0 [73.0, 83.0]	<0.001
Diabetes, n (%)		8955 (2.15)	6769 (6.49)	1121 (1.08)	886 (0.85)	179 (0.17)	<0.001
Hypertension, n (%)		233,557 (56.04)	103,870 (99.60)	101,014 (97.02)	28,673 (27.51)	0 (0.00)	<0.001
CHD, n (%)		16,598 (3.98)	8986 (8.62)	4478 (4.30)	2271 (2.18)	863 (0.83)	<0.001
TC, mmol/L		5.65 [4.91, 6.43]	5.41 [4.58, 6.27]	5.79 [5.03, 6.58]	5.76 [5.07, 6.50]	5.61 [4.96, 6.33]	<0.001
TG, mmol/L		1.49 [1.05, 2.15]	1.94 [1.40, 2.71]	1.54 [1.12, 2.16]	1.48 [1.06, 2.14]	1.11 [0.84, 1.53]	<0.001
LDL-C, mmol/L		3.52 [2.94, 4.12]	3.41 [2.76, 4.07]	3.62 [3.02, 4.23]	3.62 [3.08, 4.18]	3.41 [2.91, 3.97]	<0.001
HDL-C, mmol/L		1.40 [1.17, 1.68]	1.21 [1.04, 1.42]	1.43 [1.21, 1.68]	1.39 [1.17, 1.67]	1.59 [1.36, 1.85]	<0.001
FBG, mmol/L		4.96 [4.61, 5.32]	5.11 [4.73, 5.66]	4.98 [4.70, 5.34]	4.90 [4.58, 5.24]	4.80 [4.49, 5.11]	<0.001
HbA1c, %		5.37 [5.15, 5.62]	5.58 [5.33, 5.93]	5.37 [5.15, 5.58]	5.35 [5.14, 5.57]	5.25 [5.04, 5.45]	<0.001

BMI, body mass index; CHD, coronary heart disease; TC, total cholesterol; TG, triglycerides; LDL-C, low-density lipoprotein cholesterol; HDL-C, high-density lipoprotein cholesterol; FBG, fasting blood glucose; eGDR, estimated glucose disposal rate.

### 3.2 Association of eGDR With AAA

**Fig. 2.**
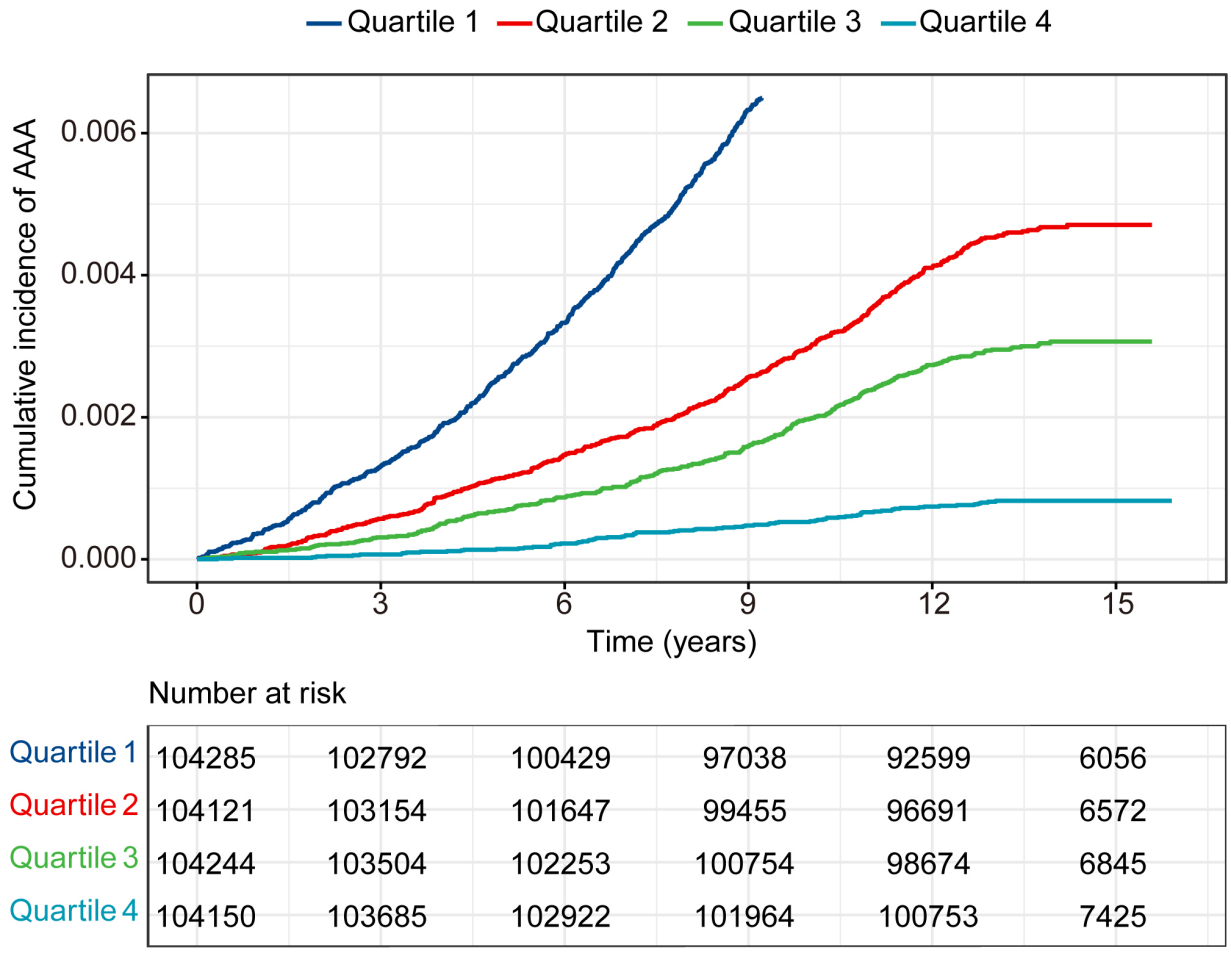
**Cumulative incidence rate of AAA in 416,800 individuals separated by the four categories of eGDR**. AAA, abdominal aortic aneurysm.

**Fig. 3.**
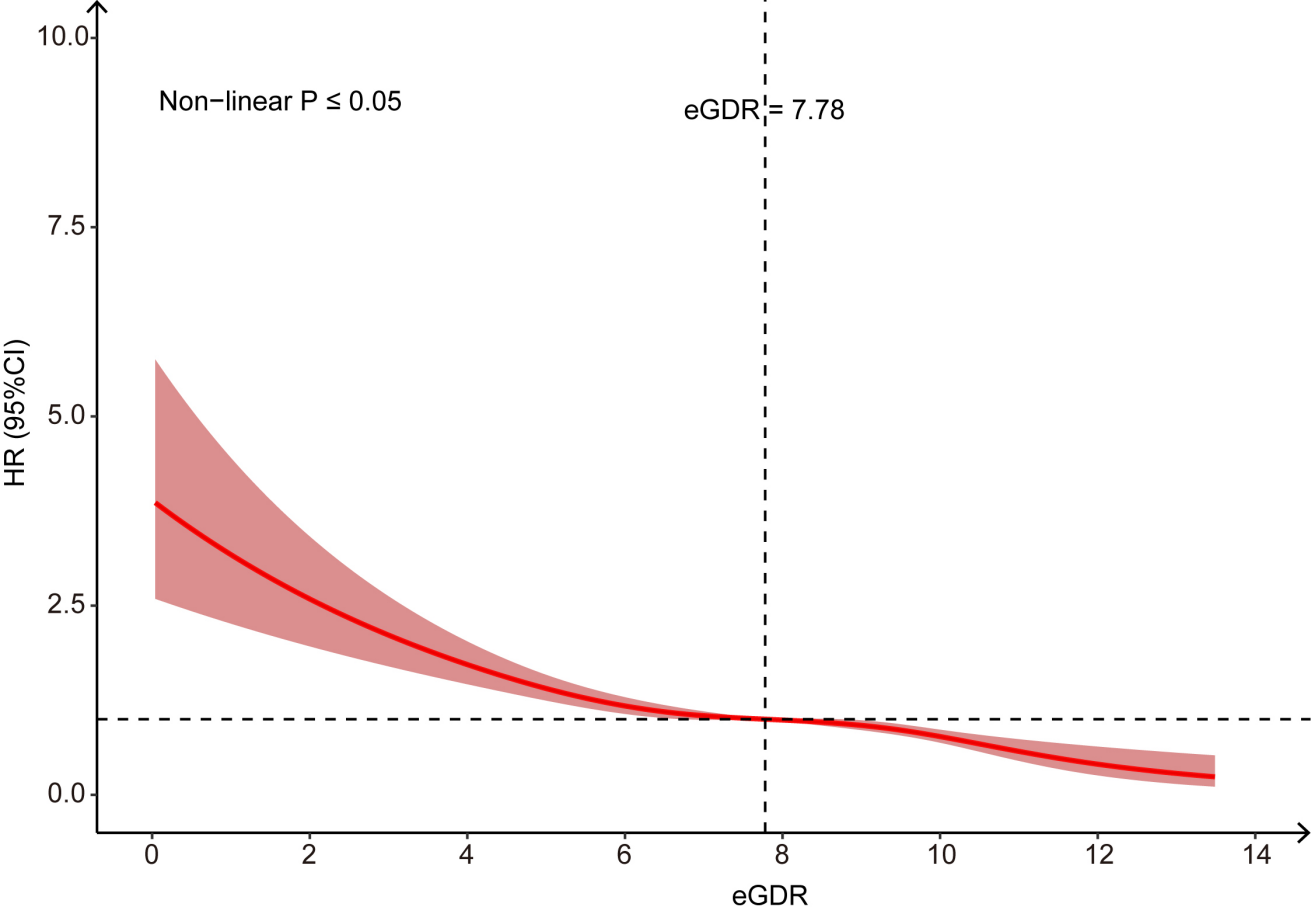
**Restricted Cubic Spline to evaluate the association between eGDR and AAA**.

Fig. 2 presents Kaplan-Meier survival curves constructed for eGDR quartiles, demonstrating significantly higher cumulative AAA incidence in the lowest versus the highest quartile. Over a median follow-up period of 13.6 years, 1881 incident AAA cases were ascertained. RCS analysis revealed a nonlinear association between eGDR and AAA risk (nonlinearity *p* ≤ 0.05), with a threshold effect observed at an eGDR value of 7.78, showing risk reduction above eGDR 7.78 mg/kg/min but accelerated risk elevation below this threshold (Fig. 3).

In categorical analyses using eGDR quartiles, progressively reduced AAA risks were observed across higher quartiles compared to Q1 (reference): the hazard ratio (HR) (95% CI) of AAA in eGDR quartiles 2 (Q2), quartiles 3 (Q3), and quartiles 4 (Q4) were 0.44 (0.39–0.49), 0.28 (0.25–0.32), and 0.08 (0.06–0.10), respectively (*p* for trend < 0.001; Table 2). This inverse association remained significant after adjusting for smoking and alcohol use (Model 2, *p* < 0.001 for trend) and additional covariates, including ethnicity, education, environment, BMI, diabetes, and glucose and lipid profiles (Model 3, *p* < 0.001 for trend). In the fully adjusted model (Model 3), Q4 participants exhibited 54% lower AAA risk versus Q1 [HR 0.46 (95% CI: 0.35–0.62), *p* < 0.001 for trend]. When eGDR was analyzed as a continuous variable, each unit increment of eGDR was associated with a 27% lower AAA risk [HR 0.73 (95% CI: 0.72–0.75), *p* < 0.001] in the unadjusted model. After full covariate adjustment, each unit increase of eGDR remained associated with a 12% lower AAA risk [HR 0.88 (95% CI: 0.85–0.90), *p* < 0.001]. Sensitivity analyses demonstrated robustness of results (Table 2). When excluding patients diagnosed with diabetes, per unit increment of eGDR was associated with 12% lower AAA risk [HR 0.88 (95% CI: 0.84–0.91), *p* < 0.001]. When excluding the population with short follow-up time, the eGDR on the risk of AAA was similar.

**Table 2.** **Baseline of eGDR and AAA incident risk**.

	eGDR, HR (95% CI)				*p* for trend	Per Union	*p*-value
	Quartile 1	Quartile 2	Quartile 3	Quartile 4			
Unadjusted model	Reference	0.44 (0.39–0.49)	0.28 (0.25–0.32)	0.08 (0.06–0.10)	<0.001	0.73 (0.72–0.75)	<0.001
Model 1	Reference	0.60 (0.54–0.67)	0.56 (0.49–0.64)	0.32 (0.25–0.40)	<0.001	0.73 (0.72–0.75)	<0.001
Model 2	Reference	0.67 (0.61–0.76)	0.60 (0.53–0.69)	0.36 (0.29–0.46)	<0.001	0.87 (0.85–0.89)	<0.001
Model 3	Reference	0.76 (0.66–0.87)	0.69 (0.59–0.80)	0.46 (0.35–0.62)	<0.001	0.88 (0.85–0.90)	<0.001
Sensitivity analysis 1	Reference	0.71 (0.61–0.83)	0.67 (0.57–0.80)	0.45 (0.34–0.61)	<0.001	0.88 (0.84–0.91)	<0.001
Sensitivity analysis 2	Reference	0.78 (0.68–0.89)	0.69 (0.59–0.81)	0.48 (0.35–0.63)	<0.001	0.88 (0.85–0.91)	<0.001
Sensitivity analysis 3	Reference	0.77 (0.67–0.89)	0.70 (0.59–0.82)	0.48 (0.36–0.64)	<0.001	0.88 (0.85–0.92)	<0.001

Model 1: adjusted for sex and age; Model 2: adjusted for sex, age, drinking, and smoking status; Model 3: adjusted for age, gender, ethnicity, education, smoking, drinking, environment, BMI, diabetes, CHD, FBG, TC, TG, LDL-C, HDL-C. Sensitivity analysis 1: excluded patients diagnosed with diabetes. Sensitivity analysis 2: excluded follow-up time less than 1 year. Sensitivity analysis 3: excluded follow-up time less than 3 years.

### 3.3 Subgroup Analysis

**Fig. 4.**
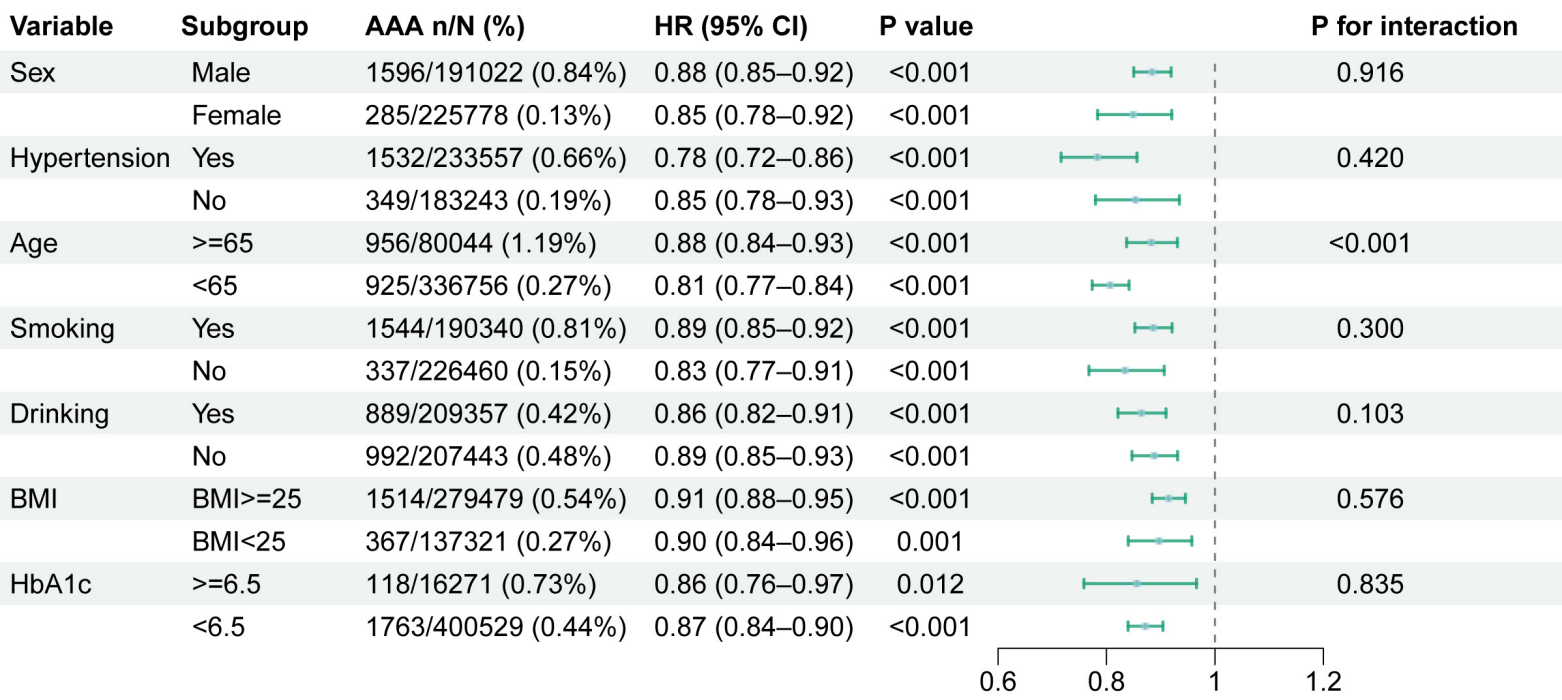
**Subgroup analysis of eGDR on AAA**.

Fig. 4 presents stratified analyses evaluating the consistency of eGDR-AAA associations across demographic and clinical subgroups. The effect of eGDR on AAA risk was consistent across all subgroups. Consistent with known epidemiology, men exhibited a significantly higher incidence of AAA (0.84%) compared to women (0.13%). Sex-stratified analyses demonstrated comparable risk reduction per unit eGDR increase: Male: (HR 0.88, 95% CI: 0.85–0.92), Female: (HR 0.85, 95% CI: 0.78–0.92). In participants with hypertension, the multivariable-adjusted HR for AAA was 0.78 (95% CI: 0.72–0.86, *p* < 0.001). Among those without hypertension, the corresponding HR was 0.85 (95% CI: 0.78–0.93, *p* < 0.001). Subgroup analysis stratified by age groups revealed a significant interaction between age and the association of eGDR with AAA risk (*p*-interaction < 0.001). Specifically, the effect of eGDR was more pronounced in participants aged <65 years (HR 0.81, 95% CI: 0.77–0.84) compared to those aged ≥65 years (HR 0.88, 95% CI: 0.84–0.93), indicating age-dependent heterogeneity in the magnitude of risk reduction. Subgroup analysis by HbA1c levels (≥6.5% vs. <6.5%) showed consistent associations between eGDR and AAA. For HbA1c ≥6.5%, HR was 0.86 (95% CI: 0.76–0.97, *p* = 0.012); for HbA1c <6.5%, HR was 0.87 (95% CI: 0.84–0.90, *p* < 0.001). No significant interaction was observed between HbA1c stratification and eGDR-AAA association (*p*-interaction = 0.835). **Supplementary Table 1** further presents characteristics stratified by diabetes status, revealing distinct metabolic patterns between diabetic and non-diabetic subgroups. The total sample size in **Supplementary Table 1** (N = 416,799) accounts for the exclusion of one participant with a missing baseline diagnosis value.

### 3.4 Additional Analysis About the Parameters of eGDR

As previously noted, the eGDR was developed as a composite parameter reflecting insulin resistance, comprising three clinical variables: waist circumference, hypertension status, and glycated hemoglobin. To investigate the relative contributions of these three components within the eGDR formula, we conducted standardized contribution assessment analyses, with results detailed in **Supplementary Table 2**. Based on the eGDR formula, HT (binary variable) had the largest absolute impact on eGDR values (–3.407 units if present). WC and HbA1c, as continuous variables, contributed less per unit change. In addition, the predictive performance of eGDR and its components for AAA risk was evaluated using receiver operating characteristic (ROC) analysis (**Supplementary Fig. 1**). The eGDR composite score demonstrated superior discriminative ability with an AUC of 0.715, significantly outperforming hypertension alone (AUC = 0.628, *p* < 0.001), waist circumference (AUC = 0.693, *p* < 0.001), and HbA1c (AUC = 0.632, *p* < 0.001). These results statistically confirm that the eGDR provides incremental predictive value compared to hypertension, waist circumference, and HbA1c.

## 4. Discussion

This study, leveraging data from 416,800 UK Biobank participants, identified an independent inverse association between eGDR and AAA incidence over a median follow-up of 14.6 years. The incidence of AAA varied across eGDR quartiles, with the highest quartile exhibiting the lowest risk. Robustness was confirmed through three prespecified sensitivity analyses. The results of subgroup analysis corroborated the main findings, further strengthening the validity of the observed associations.

### 4.1 Clinical Significance

Although the hyperinsulinemic-euglycemic clamp remains the gold standard for assessing insulin resistance, its utility in large-scale epidemiological studies and routine clinical practice is constrained by procedural complexity. eGDR has been validated as a reliable marker of insulin resistance, particularly in Type 1 diabetes [6], and also has recently been recognised as a prognostic indicator for cardiovascular outcomes and mortality [11, 14]. Zheng *et al*. [16] demonstrated an inverse relationship between long-term eGDR levels and cardiovascular risk in both European and Asian populations. An analysis of individuals without diabetes mellitus indicated that lower levels of eGDR were associated with an increased risk of cardiovascular diseases among non-diabetic participants [14]. A prospective cohort study showed that a higher eGDR level was associated with a decreased risk of cardiovascular disease in non-diabetic chronic kidney disease patients [17]. A lower eGDR was associated with a higher risk of cardiovascular diseases in prediabetic populations [18], as was also found in the American population [19]. Yi *et al*. [20] demonstrated that each standard deviation increment in eGDR was associated with a 30% reduced likelihood of developing atherosclerotic cardiovascular disease (HR 0.70, 95% CI: 0.60–0.80). We demonstrated that higher eGDR values were inversely associated with AAA susceptibility, consistent with existing evidence on cardiovascular and atherosclerotic conditions. Restricted cubic spline analysis revealed a non-linear relationship between eGDR and AAA occurrence, mirroring patterns reported in previous cardiovascular studies [16]. These findings provide novel evidence linking eGDR to AAA susceptibility, positioning eGDR as a potential marker for both insulin resistance and AAA risk prediction.

Subgroup analysis indicated that eGDR may serve as a reliable biomarker for AAA risk prediction. The higher AAA incidence in males likely reflects sex-specific biological factors (e.g., hormonal profiles, abdominal wall anatomy), yet these factors did not modify the eGDR-AAA association. Further studies could explore sex-specific pathways linking insulin resistance to vascular pathogenesis. Hypertension-stratified analyses demonstrated consistent eGDR-AAA associations across subgroups, with numerically stronger effect sizes in hypertensive individuals. The observed age-dependent heterogeneity in eGDR-AAA associations highlights age-related modifications in metabolic-vascular interactions. In younger populations, metabolic plasticity may amplify insulin sensitivity benefits, whereas vascular aging, comorbidities, and polypharmacy in older adults may attenuate these effects. Notably, consistent eGDR effects were observed across age-based AAA screening criteria, reinforcing its predictive validity. eGDR could serve as an adjunct to ultrasound screening for high-risk individuals outside standard screening age ranges. For individuals with low eGDR (indicating high insulin resistance), abdominal ultrasound or contrast-enhanced computed tomography (CT) screening may be prioritized. HbA1c-stratified analyses revealed robust eGDR-AAA associations, excluding confounding by glycemic status and enhancing result credibility.

### 4.2 Mechanism Explanation

Emerging evidence indicates that impaired insulin sensitivity plays a pivotal role in the development of atherosclerotic vascular diseases [21]. Insulin resistance disrupts glucose metabolism, inducing oxidative stress, inflammation, and vascular cell apoptosis that collectively impair endothelial and smooth muscle function [22, 23]. Beyond glucose metabolism dysregulation, impaired insulin signaling disrupts lipid homeostasis, elevating blood lipid levels that potentiate both early and advanced atherosclerotic plaque formation [24, 25]. Given the established role of atherosclerosis in AAA pathogenesis [26], the eGDR-AAA association may be mediated through these metabolic pathways. Although insulin resistance damages vascular systems through oxidative stress, inflammation, cellular apoptosis, and lipid metabolism dysregulation, direct experimental evidence linking insulin resistance to AAA remains lacking. Future molecular and animal studies are required to explore this association, including developing animal models of insulin resistance or AAA, and quantifying inflammatory biomarkers in abdominal aortic tissues.

eGDR was developed as an effective indicator of insulin resistance [6]. Insulin resistance refers to a pathological state characterized by reduced target tissue responsiveness to circulating insulin [27]. Insulin resistance contributes to the pathogenesis of multiple disorders, including obesity [28], metabolic syndrome [29], non-alcoholic fatty liver disease [30], atherosclerosis [31], and diabetes mellitus (DM) [32]. Our findings demonstrate a dose-dependent relationship between insulin resistance severity and AAA risk. Sensitivity analyses excluding diabetic participants yielded consistent results. Paradoxically, it is known that diabetic patients tend to develop smaller AAA [33]. Based on the results of previous studies, the potential explanation was given as follows: (1) Insulin resistance represents a key pathophysiological feature of diabetes mellitus, preceding overt hyperglycemia [34]. The prediabetes-AAA association remains mechanistically undefined. Diabetes progression stages may exert differential effects on AAA pathogenesis, warranting deeper investigations. Future studies should examine eGDR-AAA associations across different glycemic control states. (2) Insulin resistance and hyperglycemia mediate vascular pathophysiology through distinct molecular pathways [35]. Thus, insulin resistance and diabetes mellitus differentially influence AAA pathogenesis. (3) A population-based cohort study demonstrated that oral hypoglycemic agents reduce AAA incidence in diabetics [36]. These agents ameliorate insulin resistance [37]. Pharmacological interventions may modulate the differential impacts of insulin resistance versus diabetes on AAA. This critical knowledge gap warrants prioritized investigation.

## 5. Strengths and Limitations

Our study represents the first investigation into the association between eGDR and AAA, offering novel insights into how insulin resistance may contribute to AAA pathogenesis. The principal strengths of this investigation encompass its prospective study design, large-scale cohort, and extended follow-up period. The validity of our conclusions is supported by rigorous sensitivity analyses and subgroup analyses, which collectively enhance the robustness of our findings.

However, certain limitations should be acknowledged. (1) Selection bias may have emerged due to missing eGDR measurements in some individuals, potentially affecting our analysis of eGDR and AAA. (2) A key methodological limitation stems from the exclusive reliance on ICD-10 diagnostic codes for AAA identification, without integration of surgical records or imaging documentation, which may have led to an underestimation of the true AAA population. Future studies should incorporate multimodal diagnostic approaches (including procedural and radiological confirmation) to establish more robust validation of the eGDR-AAA risk association. (3) The median age in the population was less than the screening age of AAA. We used the subgroup analysis to deal with this problem, and more studies according to age stratification are needed to validate the relationship between eGDR and AAA. (4) The generalizability of these results requires careful consideration, as our study population consisted primarily of Western individuals aged 40 and above. Future research should expand to include more demographically diverse groups, especially including diverse racial and young cohorts, to validate these findings. (5) Another important limitation is that at baseline, diabetes was diagnosed solely through ICD-10 codes without incorporating HbA1c values as supplementary diagnostic criteria (HbA1c was established as a diagnostic criterion for diabetes in 2012 in the UK [38]), coupled with absence of diabetes staging analysis and medication usage data, potentially leading to an underestimation of diabetes prevalence and inadequately analysis about interfered factor. Although sensitivity and subgroup analyses were conducted to confirm the robustness of our findings, further research is warranted to elucidate the impact of diabetes on the relationship between eGDR and AAA. (6) Given the inherent limitations of observational designs in establishing causal pathways, we were unable to adequately control for confounding factors. Future investigations should prioritize Mendelian randomization studies or other causal inference methodologies to disentangle the bidirectional relationship between eGDR and AAA pathogenesis.

## 6. Conclusions

Our analysis establishes eGDR—a validated surrogate marker of insulin resistance—as exhibiting a significant independent association with AAA risk. These results highlight the crucial role of metabolic homeostasis and insulin sensitivity in AAA development, suggesting that targeting metabolic dysregulation may be a promising strategy for AAA prevention.

## Data Availability

This research has been conducted using the UK Biobank Resource. All bona fide researchers in academic, commercial, and charitable settings could have access to the data upon application once they meet the approval criteria for compensation (http://www.ukbiobank.ac.uk/register-apply).

## Abbreviations

eGDR, Estimated glucose disposal rate; AAA, Abdominal aortic aneurysm; BMI, Body mass index; CHD, Coronary heart disease; FBG, Fasting blood glucose; TC, Total cholesterol; TG, Triglyceride; LDL-C, Low-density lipoprotein cholesterol; HDL-C, High-density lipoprotein cholesterol; ICD-10, International classification of diseases, 10th revision; RCS, Restricted cubic spline; HR, Hazard ratio; CI, Confidence interval; HT, Hypertension; WC, Waist circumference.

## Supplementary Material

Supplementary material associated with this article can be found, in the online version, at https://doi.org/10.31083/RCM36776.
