# Supplementary material for: Association of Estimated Glucose Disposal Rate With Risk of Abdominal Aortic Aneurysm: Evidence From a Large-Scale Prospective Cohort Study of the UK Biobank
Source: Rev Cardiovasc Med. 2025 Jul 21;26(7):36776. doi: 10.31083/RCM36776 (PMC12326403; doi:10.31083/RCM36776)
Supplement: Supplementary file 1 [file 2153-8174-26-7-36776-s1.docx]

**Supplementary table 1** Baseline characteristics of participants

| Characteristics | Non-Diabetes N=407844 | Diabetes N=8955 | P value |
| --- | --- | --- | --- |
| eGDR | 7.854 [6.218, 10.392] | 4.833 [3.648, 6.109] | <0.001 |
| Age, years | 58.000 [50.000, 63.000] | 62.000 [56.000, 66.000] | <0.001 |
| Male, n (%) | 185474 (45.48) | 5548 (61.95) | <0.001 |
| Female,n(%) | 222370 (54.52) | 3407 (38.05) | <0.001 |
| Ethnic, n (%) |  |  | <0.001 |
| White | 19479 (4.78) | 976 (10.90) |  |
| Others | 388365 (95.22) | 7979 (89.10) |  |
| Education, n (%) |  |  | <0.001 |
| High | 133515 (32.74) | 1833 (20.47) |  |
| Medium | 205780 (50.46) | 4373 (48.83) |  |
| Low | 68549 (16.81) | 2749 (30.70) |  |
| Smoking, n (%) |  |  | <0.001 |
| Yes | 185215 (45.41) | 5125 (57.23) |  |
| No | 222629 (54.59) | 3830 (42.77) |  |
| Drinking, n (%) |  |  | <0.001 |
| Yes | 206591 (50.65) | 2765 (30.88) |  |
| No | 201253 (49.35) | 6190 (69.12) |  |
| Environment, n (%) |  |  | <0.001 |
| Urban | 349210 (85.62) | 8000 (89.34) |  |
| Country | 58634 (14.38) | 955 (10.66) |  |
| BMI, kg/m2 | 26.680 [24.112, 29.793] | 30.683 [27.316, 34.858] | <0.001 |
| Waistline, cm | 90.000 [80.000, 99.000] | 103.000 [93.000, 113.000] | <0.001 |
| Hypertension, n (%) | 226010 (55.42) | 7546 (84.27) | <0.001 |
| CHD, n (%) | 14199 (3.48) | 2399 (26.79) | <0.001 |
| TC, mmol/L | 5.676 [4.946, 6.442] | 4.292 [3.707, 4.978] | <0.001 |
| TG, mmol/L | 1.482 [1.046, 2.145] | 1.790 [1.220, 2.574] | <0.001 |
| LDL-C, mmol/L | 3.535 [2.971, 4.130] | 2.518 [2.112, 3.010] | <0.001 |
| HDL-C, mmol/L | 1.404 [1.177, 1.680] | 1.138 [0.960, 1.372] | <0.001 |
| FBG, mmol/L | 4.926 [4.601, 5.298] | 6.801 [5.289, 9.470] | <0.001 |
| HBAC1, % | 5.372 [5.143, 5.601] | 6.955 [6.223, 7.870] | <0.001 |

BMI: body mass index; CHD: Coronary heart disease; TC：Triglycerides; TG: Cholesterol; LDL-C: low-density lipoprotein cholesterol; HDL-C: High-density lipoprotein cholesterol; FBG: Fasting blood glucose; HBAC1: glycated hemoglobin;

**Supplementary table 2** The result of the Standardized Contribution Assessment to eGDR

| Parameter | Standard deviation (SD) | Raw Coefficient | Standardized Impact |
| --- | --- | --- | --- |
| Hypertension | - | -3.407 | -3.407 |
| WC | 13.441 | -0.09 | -1.21 |
| HbA1c | 0.616 | -0.551 | -0.34 |

eGDR = 21.158 − (0.09 * WC) − (3.407 * HT) − (0.551* HbA1c) [WC = waist circumference (cm), HT = hypertension status (1=present, 0=absent), and HbA1c = glycated hemoglobin (%)]; Standardized impact= standard error * raw coefficient.


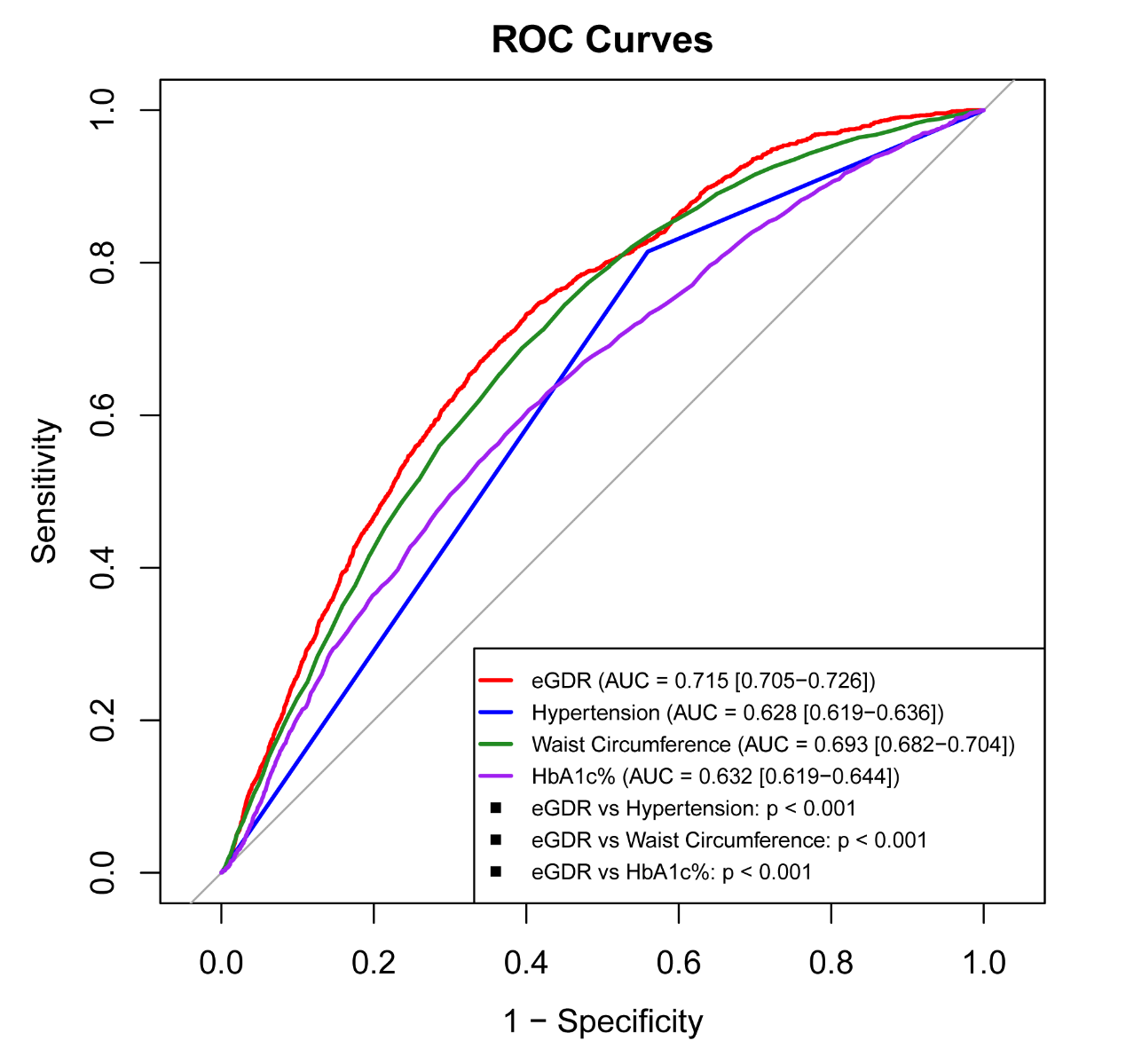


**Supplementary Fig. 1**. ROC curves of eGDR, hypertension, waist circumference, and HbA1c to predict risk of AAA. ROC: receiver operating characteristic; AUC: area under the curve; eGDR: estimated glucose disposal rate
